# L-Carnitine and Acylcarnitines: Mitochondrial Biomarkers for Precision Medicine

**DOI:** 10.3390/metabo11010051

**Published:** 2021-01-14

**Authors:** Marc R. McCann, Mery Vet George De la Rosa, Gus R. Rosania, Kathleen A. Stringer

**Affiliations:** 1 The NMR Metabolomics Laboratory, Department of Clinical Pharmacy, College of Pharmacy, University of Michigan, Ann Arbor, MI 48109, USA; mrmccann@umich.edu; 2 Department of Pharmaceutical Sciences, College of Pharmacy, University of Michigan, 428 Church Street, Ann Arbor, MI 48109, USA; mvgeorge@med.umich.edu (M.V.G.); grosania@med.umich.edu (G.R.R.); 3 Division of Pulmonary and Critical Care Medicine, Department of Medicine, School of Medicine, University of Michigan, Ann Arbor, MI 48109, USA; 4 Michigan Center for Integrative Research in Critical Care, University of Michigan, Ann Arbor, MI 48109, USA

**Keywords:** metabolomics, pharmacometabolomics, mitochondria, metabolism, acyl-carnitine, metabolic flexibility

## Abstract

Biomarker discovery and implementation are at the forefront of the precision medicine movement. Modern advances in the field of metabolomics afford the opportunity to readily identify new metabolite biomarkers across a wide array of disciplines. Many of the metabolites are derived from or directly reflective of mitochondrial metabolism. L-carnitine and acylcarnitines are established mitochondrial biomarkers used to screen neonates for a series of genetic disorders affecting fatty acid oxidation, known as the inborn errors of metabolism. However, L-carnitine and acylcarnitines are not routinely measured beyond this screening, despite the growing evidence that shows their clinical utility outside of these disorders. Measurements of the carnitine pool have been used to identify the disease and prognosticate mortality among disorders such as diabetes, sepsis, cancer, and heart failure, as well as identify subjects experiencing adverse drug reactions from various medications like valproic acid, clofazimine, zidovudine, cisplatin, propofol, and cyclosporine. The aim of this review is to collect and interpret the literature evidence supporting the clinical biomarker application of L-carnitine and acylcarnitines. Further study of these metabolites could ultimately provide mechanistic insights that guide therapeutic decisions and elucidate new pharmacologic targets.

## 1. Introduction

Mitochondria are widely investigated targets across different fields of biomarker discovery due to their expansive regulatory functions, which produce a vast range of molecules in response to disease and/or cellular dysfunction [1,2,3,4,5,6]. Many metabolites are closely tied to components of mitochondrial function, but herein L-carnitine (LC) and the acylcarnitines (ACs) will be discussed for their untapped potential to serve as biomarkers for illness and drug response, including adverse drug reactions (ADRs).

The carnitine pool, comprised of LC and the acylated derivatives (ACs), is recognized for facilitating fatty acid β-oxidation (FAO) in mitochondria and peroxisomes [7,8]. The carnitine pool represents a group of mitochondrial-derived metabolites, the blood concentrations of which generally reflect disorders of long-chain FAO, also known as the inborn errors of metabolism [9,10,11]. The Health Resources and Services Administration of the U.S. Department of Health and Human Services recommends that neonatal screening includes tests for inborn errors of metabolism such as LC uptake or transport defects, medium-chain acyl-coenzyme A (CoA) dehydrogenase deficiency, long-chain L-3 hydroxyacyl-CoA dehydrogenase deficiency, very long-chain acyl-CoA deficiency, and trifunctional protein deficiency [12]. LC and AC levels are not routinely measured outside of this neonatal screening. Consequently, the utility of LC and ACs as metabolic biomarkers has predominately centered around the implications of this screening and the significant clinical impact of found defects, which have been extensively reviewed elsewhere [13,14].

Recent advances in analytical methods coupled with the advancement of the science of metabolomics have brought LC and the acylated esters into the spotlight of biomarker discovery and research. Metabolomics is a systems biology science focusing on endogenous small molecules (<1500 Da) in a single biological sample, and pharmacometabolomics is the specific application regarding the metabolic response to drugs [15,16,17]. Targeted measurements of free LC and the differing chain length ACs in the blood have revealed metabolic perturbations in patients across a variety of diseases and have been linked to drug toxicities. These findings bring new insights into the metabolic mechanisms that underlie certain diseases and ADRs. This knowledge may lead to the discovery of novel drug targets and influence therapeutic decision-making.

This review aims to illustrate the potential of blood concentrations of LC and ACs as candidate biomarkers outside the realm of the inborn errors of metabolism and into the field of acquired metabolic disorders. As we move towards a precision medicine model for a number of diseases, there is undoubtedly interest among the scientific community to better understand LC and its esters as hallmarks of disease severity and progression, as well as the metabolic impact of pharmaceutical interventions. For this narrative review, pertinent literature was compiled from Pubmed searches using the terms “carnitine”, “acylcarnitine”, “biomarker”, “impaired fatty acid oxidation”, and “adverse drug reaction”. The disease states and drugs included in the review were the topics with the most publications using these search terms. As such, we do not address a comprehensive list of diseases or drugs that may be impacted by the carnitine pool.

## 2. Carnitine, Acylcarnitines, and Mitochondrial Bioenergetics

### 2.1. Primary Role: The Carnitine Shuttle

**Figure 1:**
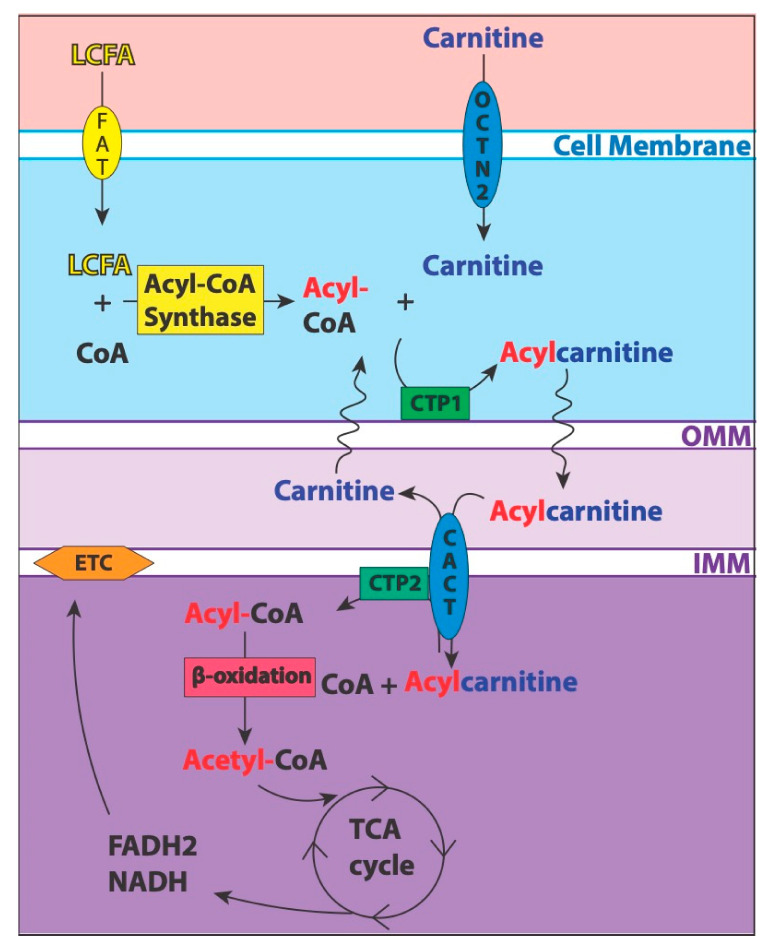
The Carnitine Shuttle. The figure highlights the major components of the carnitine shuttle system used to import long-chain fatty acids (LCFA) into the mitochondria for oxidation. LCFA are converted to acyl-CoA via acyl-CoA synthase. Then, the enzyme, carnitine palmitoyltransferase (CPT) 1, produces acylcarnitines from acyl-CoA and free carnitine. Carnitine-acylcarnitine translocase (CACT) moves acylcarnitine across the inner mitochondrial membrane (IMM) as carnitine is exported out. CPT2 converts the acylcarnitine back into acyl-CoA and free carnitine. Acyl-CoA is then available for β-oxidation that produces 1 molecule of acetyl-CoA per cycle of oxidation, which enters the TCA cycle. The cycle provides the necessary electron donors to feed into the electron transport chain (ETC), thus powering oxidative phosphorylation.FAT = fatty acid transferase, CoA = coenzyme A, OCTN2 = organic cation/carnitine transporter 2, TCA cycle = tricarboxylic acid cycle, FADH2 and NADH = electron donors, OMM/IMM = outer/inner mitochondrial membrane.

Mitochondria produce the majority of the primary energy currency, adenosine triphosphate (ATP), for the body through various oxidation pathways [18]. Of note, glycolysis and FAO are complex processes that involve a series of enzymatic reactions and translocation of intermediate products. LC participates in a shuttle system to import long-chain fatty acids into the mitochondria for subsequent FAO, as shown in Figure 1 [7,9,19].

### 2.2. Endogenous Carnitine Homeostasis

LC is a small, polar molecule de novo synthesized from two amino acids, lysine and methionine, but is largely acquired by dietary intake of animal products such as red meats, dairy, poultry, and fish [7,8]. The carnitine pool mostly resides in the skeletal muscle but is also found in the blood, liver, kidney, brain, and heart [7]. The plasma and tissue concentrations are heavily conserved, which allows for the detection of small perturbations. The normal plasma levels consist of 83% LC and 17% ACs, with acetylcarnitine (C2) representing 75% of the ACs [7]. The compounds are highly regulated through reabsorption (98%) in the renal tubules and distribution in the tissues via the sodium-dependent organic cation/carnitine transporter (OCTN) family [7,8].

Historically, LC and ACs in blood concentrations are reported differently to distinguish groups within a study. For example, they are often compared between control and experimental arms, reported as changes from baseline, or as a ratio between the summed value of all measured ACs (or individual ACs) to free LC. Since LC is much higher in abundance relative to the ACs, an AC/LC ratio in the blood exceeding 0.4 is thought to represent disturbed mitochondrial metabolism [7]. Additionally, ratios of ACs and other metabolites, such as free fatty acids, have also been used to assess different metabolic pathways [20,21,22,23].

Due to the tight regulation of the carnitine pool, perturbations in carnitine metabolism can also be identified through a quantitative, targeted analytical approach. Measurement of blood concentrations of specific ACs and LC may reveal the untapped potential of pinpointing disturbed metabolic pathways, which may help elucidate mechanisms that are affected by certain diseases and drugs.

### 2.3. Metabolic Pathways of Acylcarnitine Production

The total body carnitine pool is comprised of LC, C2, short-chain (C3–C5), medium-chain (C6–C12), and long-chain (C14–C20) ACs [7]. The conventional abbreviation for ACs is shown as C followed by the chain length number, the number of saturated bonds after the colon, DC indicates a dicarboxylic acid, and an OH represents a hydroxyl group (e.g., C16:1-OH, 16 carbons with 1 double bond and a hydroxyl attached to the acyl group).

Blood concentrations of C2 reflect intracellular levels and the regulation of acetyl-CoA and free CoA via carnitine acetyl-CoA transferase [7,24]. Increased production of C2 represents a critical mechanism for buffering the metabolic status between fed (glucose oxidation) and fasted (fat oxidation) states, referred to as metabolic flexibility [23,25]. Acetyl-CoA is a known inhibitor of pyruvate dehydrogenase (PDH), a critical enzyme involved in cellular respiration of glucose [7,26,27]. Conversely, LC has been shown to stimulate PDH in human muscle in vivo and in vitro, possibly through the reduction of acetyl-CoA (an inhibitor) by converting it to C2, thus supporting metabolic flexibility [28,29]. Therefore, persistent elevations in blood concentrations of C2 over time may represent a signal of metabolic inflexibility.

Notably, the short-chain ACs are derived from alternative energy sources, the branched-chain amino acids (BCAAs; leucine, isoleucine, and valine) [30]. During times of protein catabolism, the BCAAs concentrations can increase, prompting the utilization of LC. Specifically, propionylcarnitine (C3), C4-dicarboxylcarnitine (C4-DC), isovalerylcarnitine (C5) are produced during increased metabolism of leucine, isoleucine, and valine [24,30,31]. Peroxisomes have also been implicated in the production of varying chain length ACs including C2, C3, C6, and C8 [10,32,33,34,35]. The peroxisomes oxidize very-long chain fatty acids (>C22) and branched-chain fatty acids that are incompatible with mitochondrial enzymes [36]. Peroxisomes utilize the carnitine shuttle to transport the end-products (acetyl-CoA, propionyl-CoA, and medium-chain acyl-CoA) into the mitochondria for complete oxidation via the TCA cycle [7,37]. Peroxisomal β-oxidation is mostly involved with fatty acid biosynthesis, whereas mitochondrial β-oxidation is directed toward energy production [36]. Due to this functional distinction, mitochondrial β-oxidation likely produces the majority of the medium- and long-chain ACs measured in the plasma [24,36,38,39]. Medium- and long-chain ACs are produced when fatty acid supply exceeds demand and/or the capacity of mitochondrial β-oxidation and the TCA cycle enzymes [23].

Ultimately, the mitochondria remain the primary machinery that regulates the oxidation pathways of fatty acids, glucose, and BCAAs. Incomplete fatty acid oxidation and disruptions to glycolysis and BCAA metabolism can be assessed by measuring blood concentrations of LC and various ACs. Targeted measurements of the carnitine pool may unveil mechanistic insights that open new avenues of translational research, allowing clinicians to connect disturbances in mitochondrial metabolism to clinical phenotypes and outcomes.

## 3. Disease-Induced Alterations to Carnitine Metabolism

Disruption in mitochondrial metabolic function is attributable to a number of diseases. This has raised interest in the measurement of blood levels of LC and ACs to further understand how changes in mitochondrial macronutrient metabolism inform disease manifestation, progression, and severity. Herein we address recent findings that demonstrate how disrupted carnitine metabolism informs the phenotypes of several major diseases (see Table 1), potentially elucidating mechanistic pathways that could provide pharmacodynamic targets.

### 3.1. Diabetes Mellitus

Patients with diabetes have underlying disruptions in carbohydrate and lipid metabolism that present as elevated blood glucose levels due to impaired insulin sensitivity or production [39]. The metabolic interplay between insulin-dependent glucose metabolism and FAO are major areas of interest in diabetes research. The pathogenesis of insulin resistance and diabetes have been linked to intramitochondrial disturbances, specifically involving incomplete or reduced FAO and lipotoxicity [24,43]. Prevailing theories suggest incomplete FAO causes lipids (long-chain ACs, acyl-CoA, ceramide, diacylglycerol) to accumulate in the cytosol leading to disruption and inhibition of insulin signaling [24,39,60]. It remains unclear whether the short-chain ACs reflect or inflict insulin resistance in diabetic patients [24]. However, it is clear that C2 is involved with the regulation of metabolic flexibility since LC utilization and C2 production are important for maintaining glucose homeostasis [23]. Unchecked lipid oxidation can stifle the response to insulin and hinder the switch from lipid to glucose metabolism following a carbohydrate meal, which is likely amplified in patients with impaired insulin sensitivity. The associated increase in C2 production helps regulate this transition by reducing acetyl-CoA concentrations, thus allowing glucose oxidation to progress. Then, increases in malonyl-CoA concentrations ultimately suppress FAO through CPT1 inhibition, completing the switch from fat to glucose oxidation.

Numerous studies have reported disruptions in the carnitine pool with differential impact on levels of long- and short-chain ACs [24,43]. Type II diabetes (T2D) patients with complications (e.g., retinopathy, hyperlipidemia, neuropathy) had 25% lower serum LC levels than diabetic patients without complications [40]. Impaired insulin-dependent uptake of LC is a possible explanation; however, increased production of other ACs may also reduce the levels of LC in diabetic patients [24]. The results of this study also highlight how measurement and interpretation of specific ACs profiles can inform of specific disruptions in metabolism. For example, positive correlations between hemoglobin A1c (HbA1c), a clinical parameter of glucose control, with plasma C2 and various short- and medium-chain ACs have been reported in T2D [22,41]. A positive relationship between C2 and HbA1c suggests that patients with worse glucose control (higher HbA1) are unable to maintain glucose homeostasis despite implementation of the C2 salvage pathway. The relationship between HbA1c with the short- and medium-chain ACs indicates a global metabolic dysfunction disrupting glucose, BCAA, and fatty acid metabolism. Plasma LC, BCAA-derived short-chain (C3, C4, C4-DC, C5), medium-chain (C6, C8, C10:1), and long-chain (C14:1, C16, C18, C18:1) ACs were all significantly increased in T2D patients compared to lean, non-diabetic individuals after an overnight fast [22]. The widespread disruption to the whole carnitine pool after an overnight fast emphasizes the inability of T2D patients to switch between fuel sources compared to healthy individuals. Another study that compared insulin-resistant obese subjects and healthy lean subjects showed significantly increased concentrations of C3, C5, C6, and C8:1 in the insulin-resistant group [42]. The disruption in short-chain ACs suggests diabetes and insulin resistance also impacts the metabolism of BCAAs, which is a growing area of study in diabetes research [61].

Patients with insulin resistance and diabetes often present with perturbed long-chain ACs. The most plausible mechanism that contributes to this is intracellular inhibition/disruption of long-chain fatty acids on insulin signaling [24,39]. A prospective study published by the American Diabetes Association found that plasma levels of long-chain ACs were the most predictive of the ACs for the development of T2D [43]. Patients with gestational diabetes and newly diagnosed T2D had increased levels of serum medium-chain ACs with fewer differences in long-chain ACs levels [44]. The authors speculated that this finding indicates mitochondrial dysfunction is minimal in the early stages of T2D. Moreover, patients in the early stages of diabetes development are able to complete more cycles of FAO leading to medium-chain AC production, compared to long-term diabetic patients who succumb to FAO dysfunction (fewer cycles), and as a result produce more long-chain ACs.

These examples illustrate that measurements of LC and its derivatives can inform about the different components of mitochondrial metabolic function in diabetic patients. Associations between HbA1c and increased C2 or other ACs are indicative of global metabolic dysfunction that extends beyond hyperglycemia. Long-chain ACs seem to be the most predictive of the development of T2D; however, the medium-chain esters may also be helpful in identifying early or transient diabetes in patients. Different scenarios of LC and AC concentrations likely reflect distinct metabolic dysfunction in this population, which opens the door for new targeted therapeutic interventions or prognostic tests. Interestingly, there are a growing number of studies that show supplementation with LC improves glucose homeostasis through stimulation of the C2 salvage pathway that invokes metabolic flexibility [23,62,63]. Albeit larger studies are necessary to validate and strengthen the biomarker potential and the therapeutic benefits of targeting these pathways before translation into clinical practice is attainable.

### 3.2. Sepsis and Septic Shock

Sepsis is increasingly recognized for the metabolic derangement that develops during the progression of the disease [15,64]. Broadly, sepsis is a life-threatening organ dysfunction caused by a dysregulated host response to an infection [65]. Septic shock is the more severe form of sepsis that includes persistent hypotension and hyperlactatemia [65]. Mitochondrial metabolic dysfunction is inextricably linked to the hypermetabolic state that has been characterized in clinical and preclinical studies of sepsis [15,64,65,66,67]. In fact, it has been implicated as one of the potential causes of sepsis-induced organ dysfunction, a driving factor of mortality rates in this population [66,68]. Catabolic processes break down skeletal muscle and adipose tissue in sepsis and septic shock for subsequent energy production so metabolic flexibility may be an important patient characteristic for sepsis survival as well as a means to mitigate organ dysfunction/failure and long-term consequences of the illness [15,69]. Maintaining normal carnitine homeostasis is vital for mounting an immune response, especially in the case of severe infection such as sepsis [70]. The connection between the carnitine pool and sepsis via the immune system and metabolism makes a targeted analysis of these compounds a valuable pursuit.

Several studies have observed a range of sepsis-induced disruptions in carnitine metabolism between survivors and non-survivors of sepsis and septic shock [45,46,47,48,49]. A comprehensive study by Langley et al. found the short- and medium-chain AC plasma profiles to be the most pronounced between sepsis survivors and non-survivors at 28 days [45]. The ACs (C2, C5, C6, C8, C10) were significantly increased in the non-survivors even after adjusting for renal function. The absence of the long-chain ACs from these findings point to a similar conclusion found in some of the diabetes studies. Increased short- and medium-chain esters may reflect newly developed FAO dysfunction, meaning that β-oxidation cycles successfully shortened the long-chain fatty acids before the eventual disruption. These findings were further corroborated by another study that reported disruptions in the short- and medium-chain plasma AC profiles of sepsis patients [46]. The authors noted increased levels of various short- and medium-chain ACs were associated with markers of hepatic and renal function and infection/inflammation. However, only C2 was associated with all of these indices and 28-day mortality. The distinction separating C2 from the other ACs in this study poses an interesting hypothesis. The higher plasma C2 levels in the non-survivors compared to the survivors might be due to the unsuccessful switch between nutrient sources leading to the overproduction of C2 in an attempt to achieve metabolic flexibility. The ability or inability to switch between fuel sources during the high metabolic demands of sepsis could influence the immune response to the infection, organ dysfunction, and ultimately, survival. Puskarich et al. found distinct LC and AC serum profiles in septic shock patients between 28-day survivors and non-survivors at baseline and following supplementation with intravenous LC [47]. This finding suggests that increased concentrations of LC, C2, C3, and C8 at baseline are predictive for sepsis mortality. This conclusion also corroborates the findings of the previously mentioned sepsis studies that showed sepsis-induced perturbations in the short- and medium-chain ACs. Interestingly, the researchers also hypothesized that the use of LC supplementation provoked a latent phenotype in the metabolome, including the carnitine pool, that informed the response to the therapeutic intervention [17].

Indeed, the carnitine pool profile is informative of the metabolic derangement that presents during sepsis. The short- and medium-chain ACs appear to be the more prominently disturbed esters in this disease state. The presence of the short-chain ACs could indicate greater oxidation of the BCAA, whereas the medium-chain compounds may reflect disturbed peroxisomal FAO or partially impaired mitochondrial FAO. Regardless, targeted measurements of the carnitine pool offer insights into the metabolic derangement of sepsis that provide opportunities to develop new pharmacologic interventions and prognostic biomarkers.

### 3.3. Cancer

Reprogrammed metabolism, a hallmark characteristic of cancer, provides the necessary conditions for growth in substrate-dependent environments [71]. The Warburg effect, which favors glycolysis, leads to an upregulation in the compensatory pathways to fuel the TCA cycle, specifically, increased oxidation of BCAAs and fatty acids [72]. Considering these adaptations, the carnitine pool is uniquely positioned to reflect the changes that occur during cancer development and progression. Targeted measurement of LC and ACs may afford new opportunities in cancer diagnosis, prognosis, and even new pharmacological targets. Herein, we discuss the forms of cancer for which there is evidence of disrupted carnitine metabolism.

Hepatocellular carcinoma (HCC) is primarily caused by chronic inflammation and liver damage stemming from various insults such as hepatitis B, hepatitis C, and nonalcoholic fatty liver disease (NAFLD) [73]. The liver is a major regulator of energy metabolism and the primary location of LC biosynthesis [73,74]. In cases of severe liver damage, such as HCC, there are reports of impaired FAO leading to elevated ACs in the blood [50,73]. Several studies have reported similar findings about the ability of serum concentrations of LC, short-chain, medium-chain, and long-chain ACs to differentiate patients with HCC from those with liver disease or healthy controls. The authors observed increased LC, decreased short- and medium-chain ACs, and increased long-chain ACs [50,51,52,53]. Differences in the AC response to disease can inform the metabolic landscape without the need for an invasive measurement such as a liver biopsy. The distinct pattern of decreased short- and medium-chain esters with increased long-chain ACs indicates the disruption is likely early in FAO. The increase in LC could represent a number of metabolic impairments such as reduced uptake of LC or cell death leading to an intracellular LC contribution to the blood level. Based on the available studies, measured carnitines may offer a diagnostic tool to distinguish HCC patients from healthy subjects or those with other liver diseases.

Irregularities in AC concentrations have been identified in other cancers as well. A metabolomics analysis conducted using the plasma of breast cancer patients with matched controls found an association between C2 and the risk of breast cancer [54]. It logically follows that cancer presenting in different tissues would have differing effects on carnitine metabolism. The signal in breast cancer patients pointing to C2 as the lone AC suggests the malignant tissue invokes the metabolic flexibility pathway as it switches between glucose and fat utilization. Another metabolomics study aimed to discriminate colorectal adenoma, colorectal cancer, and healthy subjects [55]. The authors found that serum ACs spanning the whole spectrum from short-chain to long-chain as consistently differentiated between the patient groups. The findings suggest that ACs may have value as early detection biomarkers of colorectal adenoma and colorectal cancer. Yet another metabolomics analysis in serum concluded differences in carnitine metabolism between papillary thyroid cancer and benign nodules [56]. Yao et al. observed increased serum concentrations of medium- and long-chain ACs in the cancer cohort compared to the benign group. In line with previous conclusions, the increase in medium- and long-chain ACs suggests that patients are in an early to mid-stage of cancer development. This type of inferencing, if further studied and properly validated, could add new criteria to the staging of cancers, which could significantly influence therapeutic decision-making.

The prolific metabolic switching observed in various cancers emphasizes the crucial role metabolic flexibility plays in this disease, which is reflected in the blood LC and AC profile [75]. The diversity of cancer is vast, manifesting in different tissues through various mechanisms, yet current evidence suggests that differential concentrations of ACs may have diagnostic value for certain forms. By measuring the carnitine pool, clinicians and researchers can identify pathways that are exploited by cancer cells, which could lead to new biomarkers or druggable targets.

### 3.4. Heart Failure

Due to the advancements in cardiovascular treatments and increased life span of the aging population, a paradoxical increase in patients with heart failure is occurring [76,77]. In these patients, dysfunctional myocardial tissue has known energy disruptions and shortcomings that include decreased ATP and phosphocreatine, as well as, abnormalities in FAO [76]. Considering this disruption, the carnitine profile represents measurable metabolites in the blood that directly reflect the metabolic derangement present in the failing heart [78]. Additionally, the healthy heart primarily uses FAO for energy, but showcases metabolic flexibility by utilizing glucose, lactate, ketone bodies, and amino acids, further supporting the utility of the carnitine pool in understanding the metabolic consequences of heart failure.

A recently published study reported that plasma long-chain ACs were significantly elevated in patients with heart failure compared to controls [57]. Furthermore, the elevated AC signal (C16, C18:2, C18:1, C16:1-OH/C14:1-DC, C20:4) increased linearly with decreasing left ventricular ejection fraction, which is a defining clinical characteristic of heart failure. The signal enabled the authors to distinguish between the two heart failure phenotypes since the long-chain ACs were higher in those with reduced ejection fraction (HFrEF) compared to the subjects with preserved ejection fractions (HFpEF). The phenotypic distinction suggests the ACs have potential utility in supporting the current heart failure classification as an objective measure of heart failure staging but would need further replication and validation. A more clinically guided study by Ahmed et al. identified several groups of plasma ACs that were associated with defined clinical outcomes [58]. Groups of mostly medium- and long-chain ACs were found to be associated with all-cause mortality or all-cause hospitalization when adjusted for known predictors of each outcome. However, only the long-chain AC group (C16, C18:2, C18:1, C18, C20:4) was associated with lower peak VO_2_ (a negative clinical characteristic of heart failure), cardiovascular death/hospitalization, and heart failure exacerbation. Furthermore, the individual levels of C16, C18:1, and C18:2 were significantly higher in patients with end-stage heart failure prior to left ventricular assist device implantation and decreased with improved circulatory support. The findings of this study suggest long-chain ACs have clinical utility in assessing disease severity in the heart failure population. After validation in larger cohorts, clinicians could eventually personalize therapy based on a given patient’s AC profile. A metabolomic analysis conducted by Ruiz et al. found that plasma LC and numerous ACs differentiated between a healthy control group and patients with heart failure [59]. The heart failure patients had higher levels of LC and various chain-length ACs (C2, C4, C6, C8, C10, C12, C14, C16, C18, C18:1, C18:2) than in the control group, when adjusted for sex, age, renal function, and insulin resistance. Additionally, C2 and the medium-chain ACs were positively associated with NT-ProBNP, a clinical marker of disease severity, suggesting that the increase in metabolite concentrations were indicative of worsening disease. Interestingly, the researchers stratified the heart failure group by diabetes status and found C2 to be increased in the diabetic subgroup. The full range of ACs reflects the widespread disruption to FAO, as well as BCAA and glucose oxidation that occurs in heart failure. However, the C2 distinction by diabetes status likely reflects that patients with heart failure have more disruptions in FAO than glucose metabolism. This is further supported by other studies predominantly showing the long-chain ACs as the differentiating ACs in heart failure.

Of all the ACs, the long-chain ACs seem to be the most associated with heart failure clinical outcomes. They also may play a physiological role in exacerbating disease progression. The known biological effects of long-chain ACs suggest a possible mechanism of inflicting cellular damage through promoting skeletal muscle inflammation, reactive oxygen species (ROS) production, cellular stress, and insulin resistance [57]. More research in this area is warranted to strengthen the prognostic usefulness of long-chain ACs and to further elucidate the perturbed mitochondrial pathways as potential therapeutic targets.

## 4. Drug-Induced Alterations to Carnitine Metabolism

Adverse drug reactions (ADRs) are defined by the World Health Organization (WHO) as “a response to a drug that is unintended and occurs at doses normally used in man for the prophylaxis, diagnosis or therapy of disease, or for modification of physiological function” [79]. ADRs often result from unwanted drug interactions unrelated to the drug’s intended, primary mechanism of action, and many are toxicological manifestations of off-target drug interactions with mitochondria [80,81]. These interactions result in metabolic stress, which can be toxicologically reflected in lack of drug efficacy or in a drug-related pathology.

There are different mechanisms by which a drug can impact mitochondrial function; however, this review will focus on drugs attributed to disruptions in mitochondria metabolism, specifically LC and ACs. Severe impairment of mitochondrial FAO is associated with an accumulation of fatty acid derivatives such as ACs, acylglycine esters, and dicarboxylic acids in plasma and urine [82]. Specifically, this section will discuss how the resulting changes in LC and/or AC levels after drug administration can be used as indicators of these metabolic disruptions, thereby providing a new method for therapeutic drug monitoring. This section will focus on the six drugs shown in Table 2 that are known to cause alterations in the metabolic function of the mitochondria but are not representative of all drugs with known mitotoxic effects. The specific effects of these drugs on the carnitine pool are characterized in Table 3.

### 4.1. Valproic Acid

Valproic Acid (VPA) is a widely used antiepileptic agent to treat myoclonic, atonic, and absence seizure disorders in children and adults [102,103]. VPA is generally well tolerated and safe relative to the conditions it is used to treat. Some adverse events include gastrointestinal disturbances, sedation, transient effects on coagulation, and hepatotoxicity [102]. VPA-induced hepatotoxicity can be characterized into four distinct subtypes: (1) a transient elevation in liver transaminases, (2) reversible hyperammonemia, (3) toxic hepatitis, and (4) a Reye-like syndrome [91,104]. The exact mechanism of VPA hepatotoxicity is unknown, but different theories have been proposed and three of these are related to VPA and LC [91].

**Figure 2:**
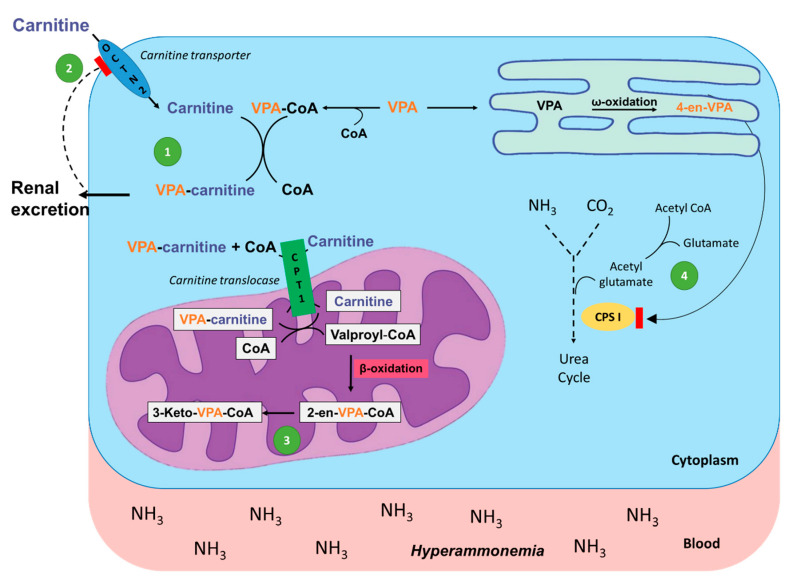
Mechanism of valproic acid (VPA)-induced hyperammonemia. (**1**) VPA is attached to coenzyme A (CoA) and transferred to LC creating VPA-carnitine, which is then renally excreted. (**2**) VPA-carnitine inhibits an ATP-dependent carnitine transporter, effectively blocking LC entry into the cell. (**3**) Other VPA metabolites, 2-en-VPA and 3-Keto-VPA, trap mitochondrial free CoA so that it cannot participate in ATP production, further inhibiting the ATP-dependent carnitine transporter. (**4**) The decrease in free CoA and LC results in decreased formation of N-acetylglutamate, which is a necessary co-factor for carbamoylphosphate synthetase I (CPS I), the primary enzyme of the first steps of the urea cycle. This leads to the accumulation of ammonia as it can no longer be incorporated into urea and excreted, resulting in hyperammonemia.

A serious consequence of VPA administration is the depletion of body LC storage. Since VPA is a short-chain fatty acid that requires LC for oxidation, it combines with LC within the mitochondria via carnitine acyltransferases. The resulting ester, valproylcarnitine, is then transported out of the mitochondria and eliminated in the urine, thus depleting body LC stores [91,105]. Carnitine deficiency due to VPA is the proposed mechanism for hyperammonemia and the development of VPA-induced hyperammonemic encephalopathy (VHE), as shown in Figure 2 [106]. This occurs when the VPA metabolites trap mitochondrial free CoA so that LC cannot be restored through the action of CPT II. The reduction of LC from this mechanism then results in decreased β-oxidation of VPA and shifts the metabolism toward ω-oxidation, producing a toxic metabolite 2-propyl-4-pentenoic acid (4-en-VPA). This metabolite reduces ammonia elimination through inhibition of carbamoyl-phosphate synthase I, the first enzyme in the urea cycle, resulting in increased ammonia levels [107].

There are several studies on the impact of VPA on carnitine levels both in vitro and in vivo [89,90,108]. One of these studies measured the concentration of LC in serum, red blood cells, muscle, liver, and urine in VPA-treated rats. The study found that the mean serum and muscle LC concentrations decreased and the mean pooled AC concentration increased relative to control animals [89]. Another study found that after injecting mice with VPA, hepatic concentrations of free CoA and LC decreased, and the ratio of AC/LC increased [90]. The authors also observed significantly increased medium-chain ACs and decreased long-chain ACs in the plasma of the VPA treated mice. These changes in the LC levels observed in rats and mice are in agreement with the aforementioned VPA mechanism that leads to LC depletion, and the consequential increase in the AC/LC ratio reflecting mitochondrial dysfunction.

In humans, a study by Opala et al. assessed the effect of VPA and other antiepileptic drug (OAD) treatments on the plasma carnitine pool in pediatric patients [91]. Both, the VPA monotherapy and polytherapy groups, had significantly lower LC levels compared to the healthy controls. The AC/LC ratio was also higher than in the VPA monotherapy and polytherapy groups when compared to the control subjects. The authors observed that the carnitine pool of the OAD (polytherapy with no VPA) group did not differ from the healthy subjects, which reinforces the theory that VPA depletes the carnitine pool.

It seems clear that VPA toxicity may be identified by measuring the disturbances of the carnitine pool. Unfortunately, LC and ACs are not routinely measured, other than in neonatal screenings as previously stated. By having information about specific levels of the ACs and the AC/LC ratio could provide critically important information and a more individual picture of each patient that could impact clinical decision-making process about prescribing, dosing, and monitoring VPA. Including routine monitoring of LC and ACs levels as part of the VPA treatment could serve as early indicators of ADRs and aid in the prescription of LC supplementation as a means to mitigate VPA-induced carnitine deficiency.

### 4.2. Clofazimine

Clofazimine (CFZ) is a FDA-approved, weakly basic, red-pigmented, phenazine antibiotic that is included in the WHO List of Essential Medications as part of the standard treatment for leprosy [109,110]. CFZ is highly lipophilic and is characterized by an unusually long pharmacokinetic half-life of up to 70 days, which is associated with extensive accumulation of the drug in the body [110,111,112]. While CFZ is well tolerated, it imposes a considerable metabolic burden and interferes with mitochondrial function [113,114,115]. CFZ is known to compromise cell viability and exert cytotoxicity by interfering with the mitochondrial ETC [112].

In a study of the metabolic consequences of chronic (8-week) CFZ administration in mice, LC was found to be one of many metabolites associated with one-carbon metabolism that was altered by CFZ treatment [92]. Notably, LC was found to be highly elevated in the animals that exhibited the most severe metabolic disruption in response to drug treatment. Furthermore, LC was the only urine metabolite that decreased after 2 weeks of CFZ treatment and remained low in the metabolically stressed animals, one of the earliest detected change in metabolism [92].

The CFZ-induced changes in LC levels can be explained, in part, by the requirement of methionine, lysine, and ascorbate for the *de novo* synthesis of LC, which is a primary source of LC in mice. Since CFZ treatment also caused a modest reduction in body weight despite an increase in the amount of food consumed, disruption in carnitine homeostasis could also have occurred by an enhanced utilization of fat stores for energy production [92].

CFZ has a known influence on mitochondrial metabolism because it accumulates within the organelle, so it is not unexpected that long-term exposure resulted in perturbed LC concentrations in mice [116]. There are no known clinical studies of CFZ treatment and metabolism. However, the CFZ-induced perturbation in mice may be useful to better understand the mechanism of CFZ associated toxicity. Moreover, monitoring of LC levels in CFZ-treated patients may prove to be useful as an indicator of pending mitotoxic effects of the drug. This could be especially useful when initiating CFZ treatment and monitoring its therapeutic effect.

### 4.3. Zidovudine

Zidovudine (ZDV) is a nucleotide reverse transcriptase inhibitor (NRTI) used in the treatment of HIV as an integral component of highly active antiretroviral therapy [117]. ZDV undergoes intracellular triphosphorylation and inhibits viral replication by incorporating into the viral DNA strand, thus impeding the viral RNA-dependent DNA polymerase, also known as reverse transcriptase. The clinical utility of many NRTIs, like ZDV and stavudine, is constrained due to their association with ADRs during chronic therapy at high doses. They have been found to gradually reduce mitochondrial function in various tissues by preventing mitochondrial replication via inhibition of the polymerase that replicates mtDNA [117,118,119].

Several in vitro and in vivo studies have alluded to possible mechanisms that contribute to ZDV-induced myopathy through the impairment of skeletal muscle mitochondria. Data suggest that the mechanism of ZDV-induced mitochondrial toxicity may be caused by carnitine deficiency [117]. A ZDV-induced reduction in the amount of LC may be a major factor in mitochondrial alterations that lead to the accumulation of lipid droplets in the cytoplasm of muscle cells [93,94,117]. These findings have been corroborated in an in vitro study in which LC treatment of C2C12 cells, a myoblastic cell line, prevented the dose-dependent ZDV-induced inhibition of cell growth [93]. These data suggest that lipid accumulation is due to depletion of LC rather than mtDNA depletion or other forms of mitochondrial dysfunction. The study also investigated the mechanism by which ZDV treatment leads to cellular reduction in LC. It was shown that ZDV reduced the transport of LC across the plasma membrane by noncompetitive inhibiting the sodium-dependent transport of LC. These data suggest that ZDV may directly interact with OCTN2, the primary carnitine transporter, interfering with the necessary renal reabsorption of LC [7,93]. This mechanism would potentiate total body carnitine deficiency and warrants further pharmacometabolomic study to confirm.

A clinical study of patients with ZDV-induced myopathic symptoms of varying severity concluded that ZDV-induced muscle mitochondrial impairment resulted in a reduction in muscle LC levels, most likely due to a decrease in LC uptake by the muscle [94]. There are two possible explanations for the decrease of LC in the muscle: (1) reduced energy within the muscle cell can cause a shift toward the glycolytic pathway that results in excess cytoplasmic acetyl-CoA. This may esterify LC, which is then exported out of the muscle and is eliminated in the urine. The other being (2) muscle uptake of LC from the blood is compromised by a shortage of energy within the cytosol due to deficiency of the mitochondrial respiratory chain enzymes, cytochrome c oxidase and reductase [94]. Both scenarios reveal that reduced muscle LC caused by mitochondrial impairment results in a shortage of energy of the muscle fibers, which manifest clinically in different degrees of myopathy and muscle weakness.

In aggregate, studies to date demonstrate that ZDV causes dysregulation of the carnitine pool leading to reduced intracellular levels. The studies above focused on skeletal muscle, but this dysregulation in carnitine metabolism may be found in other tissues and cause other ZDV-induced ADRs such as hepatic lipid accumulation (hepatic steatosis). This poses a major clinical concern for a medically vulnerable population such as HIV patients. Tracking the blood carnitine pool, and additionally muscle carnitine levels, may be an important monitoring parameter. It would permit the early identification of metabolic descent before it presents clinically, as myopathy, a known ADR of ZDV. This could also lead to the early use of LC supplementation to ameliorate myopathy symptoms.

### 4.4. Cisplatin

Cisplatin (CSP) is an effective anticancer drug used for the treatment of testicular, lung, and ovarian cancers. Although it is well-tolerated, the clinical use is limited by its side effects; nephrotoxicity, cytotoxicity, and cardiomyopathy [120]. In recent years, several studies have demonstrated that CSP-induced cytotoxicity is closely related to increased reactive oxygen species (ROS) generation and that this increase alters the mitochondrial membrane potential (MMP) and damages the respiratory chain, which ultimately triggers the apoptotic process [121,122,123].

It is well documented that CSP therapy is associated with increased excretion of a number of vital endogenous substances including LC [95,96]. A study of CSP on plasma LC concentration and urinary excretion showed a 30% increase in total carnitine plasma concentration upon treatment that normalized within 7 days after cessation of treatment [95]. These changes were associated with a tenfold increase in renal LC excretion. Similar findings were made in other studies, one of which was conducted in pediatric cancer patients [96,124]. In the pediatric cohort, there was a strong inverse correlation between fatigue and blood LC levels after a week of chemotherapy. In aggregate, CSP-induced derangement in LC was attributed to a combination of cytotoxicity that caused a release of LC from damaged tissues, and impaired renal reabsorption mechanisms, as well as inhibition of the cellular uptake of LC caused by a change in OCTN2 activity.

The use of CSP is well known for its associated toxicities, some of which have dose-limiting implications. The use of pharmacometabolomics as an approach to monitor changes in the carnitine pool could potentially help bring about a precision medicine strategy to guide CSP dosing or even therapeutic changes, especially when attempting to control fatigue after chemotherapy. Longitudinal monitoring of urine LC levels could aid in the proper use of LC supplementation to reduce ADR severity.

### 4.5. Propofol

Propofol (2,6-diisopropylphenol) is a c-aminobutyric acid (GABA) receptor agonist that is supplied as an injectable lipid emulsion for intravenous use [87]. It has become the most commonly used intravenous anesthetic due to its favorable pharmacokinetic and pharmacodynamic profile [125,126]. Propofol has been associated with a number of serious ADRs such as metabolic acidosis, cardiac asystole, myocardial failure, rhabdomyolysis, and death [127,128,129]. Propofol infusion syndrome (PRIS) is defined as metabolic acidosis (lactic acidosis) with a base deficit > 10 mmol/L in at least 1 occasion, arrhythmias, heart failure, renal insufficiency, hepatomegaly, and rhabdomyolysis following the infusion of propofol [130,131].

There is in vitro evidence that suggests impaired mitochondrial function is a mechanism by which PRIS syndrome occurs [97,130,132,133]. Studies on isolated rat liver mitochondria have demonstrated an uncoupling type of effect of propofol on mitochondrial function, which may be the result of an increase in proton permeability of the inner mitochondrial membrane [134,135]. Another study suggests that ATP production may be impaired by propofol [136]. Propofol was also found to impair the mitochondrial ETC in isolated heart samples from guinea pigs [132].

When looking at the effects of propofol and PRIS in humans, various pediatric case studies have shown changes in serum and plasma levels of ACs. A study of a child sedated with propofol reported raised serum levels of C3-DC, C5, creatine kinase, troponin T, triglyceride, lactate, and myoglobinaemia [131]. Propofol caused a disruption of mitochondrial fatty-acid oxidation and long-term infusion was associated with an increase in C3-DC, which inhibits CPT I, a critical enzyme involved with the carnitine shuttle. Consequently, the entry of long-chain AC esters in muscle tissue is impaired. Similarly, a case study with a propofol sedated patient that developed various ADRs exhibited abnormality in AC metabolism [98]. Plasma samples revealed increased levels of acetyl and hydroxyl-butyryl species, an elevation of fatty AC intermediates, especially medium-chain unsaturated and dicarboxylic species. In a third case study, where propofol infusion was administered to treat recurrent seizures, daily plasma samples showed that as the propofol infusion continued daily, C4 rose significantly above the normal range (1.0 µmol/L) [99].

In the above-mentioned cases, it is hypothesized that propofol interferes with the diffusion of medium- and short-chain fatty acids into the mitochondria and inhibits the respiratory chain (at complex II), resulting in a rise in blood levels of C5, C4, and/or C2 [97,98,99]. Based on these studies, elevated C2, C3-DC, C4, or C5 imply impaired metabolism of BCAAs and/or metabolic inflexibility, therefore, supporting their use as potential early biomarkers of the onset of PRIS that could be incorporating into a therapeutic monitoring strategy for propofol [97,98,99,131].

### 4.6. Cyclosporine

Cyclosporine (CyA) is used as an immunosuppressive agent following organ transplantation and as treatment of several autoimmune diseases [137]. Its use is limited due to its associated side effects, especially nephrotoxicity but it remains widely used as an immunosuppressant. Animal studies suggest that ROS is implicated in chronic CyA nephrotoxicity. CyA increases ROS generation and lipid peroxidation in renal tissue affecting renal function and favoring interstitial fibrosis [138]. CyA produces an increase in ROS within the mitochondria, leading to inner membrane cardiolipin oxidation and impairment of the membrane potential [139].

A study of long-term (20 weeks) CyA treatment assessing urine, blood, liver, kidney, and pancreatic concentrations of acid-soluble LC in diabetic rats found that CyA treatment caused changes in LC and myo-inositol concentrations in biologic fluids and certain tissues [100]. Diabetic rats excreted significantly higher concentrations of LC in the urine, except when treated with CyA. CyA prevented the urinary loss of LC, which caused a decrease in the measured levels of LC in urine. Similarly, CyA treatment significantly decreased LC levels in the blood. Conversely, CyA treatment resulted in an increase on the hepatic concentration of LC but did not affect the LC levels in the pancreas or kidney. A study with cadaveric kidney transplant patients being administered either azathioprine (AZA) or CyA showed significantly elevated levels of total carnitine, LC, and short-chain ACs in serum when patients were treated with CyA and prednisone and compared to AZA-treated [101].

Treatment with CyA induced changes to LC and short-chain ACs, which may suggest a drug-induced effect on the reabsorption and/or BCAA metabolism. While future studies are warranted to interrogate the mechanisms to pinpoint the specific metabolic pathways involved, it is clear that measurements of LC and short-chain ACs could provide information on the status of mitochondrial function of CyA-treated patients.

## 5. Conclusions

The paradigm shift toward precision medicine is highly dependent on the discovery, validation, and implementation of biomarkers [140]. The results presented in this review illustrate that LC and AC concentrations have significant biomarker potential across different fields of study, as summarized in Table 1 and Table 3. By measuring the carnitine pool in the blood, specific mitochondrial disruptions have been uncovered revealing various metabolic shortcoming in diabetes, sepsis, cancer, and heart failure. Interpretations of the carnitine concentration profiles could provide diagnostic and prognostic opportunities, further the knowledge of the metabolic underpinnings, and afford new therapeutic targets in these disease states.

Likewise, targeted measurements of carnitine and ACs may be informative for the identification of metabolic ADRs of drug-induced mitochondrial dysfunction during treatment with a number of different drugs including the examples discussed in this review. Drug-induced mitotoxic signals that could be detected in advance of a clinical phenotype may be particularly useful for therapeutic drug monitoring and the avoidance of an ADR altogether. In some instances, there may be therapeutic utility for the use of supplemental LC to prevent or mitigate ADRs.

We acknowledge the limitations of using metabolite concentrations as clinical biomarkers. First, with the exception of screening for inborn errors of metabolism and the assessment of carnitine deficiency, assays for ACs are not routinely available or used for clinical decision-making. Nevertheless, the status of the carnitine pool is becoming more clinically relevant and as the field advances, it is possible the clinical assays will emerge. In considering the clinical utility of AC concentrations, patient-specific factors such as diet, weight, sex, and age will need to be considered. The use of LC supplementation for the treatment of metabolic diseases or mitigating ADRs also has limitations. Rigorous efficacy and dose-ranging studies are lacking but are warranted in order to establish evidence-based use and dosing guidelines. Some studies with promising results have already been conducted in diabetes and septic shock [23,141,142]. Although LC and the AC blood concentrations will likely change with supplemental LC dosing, advancing the understanding of the extent of these changes and the factors that drive them could actually aid in furthering the biomarker capabilities and utility of LC and ACs following LC administration. In fact, this concept of using LC supplementation to provoke a latent metabolic phenotype has been explored in septic shock patients [17]. In related work, supplemental LC led to a broad dynamic range in LC and AC blood levels, with consistently higher ACs in non-survivors compared to survivors [47].

In aggregate, there is a growing body of evidence demonstrating that the measurement of LC and ACs in the blood and other biological samples have utility in identifying metabolic disorders and ADRs, but more robust, translational work is needed to bring these strategies into clinical practice. Future studies could build from the groundwork that has been laid by the current literature by reproducing and validating findings in larger, more diverse patient populations.

## Figures and Tables

**Table 1:** Examples of diseases that influence blood levels of carnitine and acylcarnitines.

Disease	Proposed Mechanism	Subject	Biospecimen	Disease-Induced Alterations in Carnitine/Acylcarnitine Levels
Diabetes Mellitus	Invoked metabolic flexibility pathway Impaired insulin-dependent uptake of carnitine Increased production of ACs due to incomplete FAO	1. Humans (T2D+complications)	1. Serum	1. 25% lower LC levels [40]
		2. Humans (T2D)	2. Plasma	2. Increased C2, SCAC, MCAC in patients with higher HbA1c [41]
		3. Humans (insulin resistant/obese)	3. Serum	3. Increased C3, C5, C6, C8:1 [42]
		4. Humans (T2D)	4. Plasma	4. Increased SCAC, MCAC, LCAC in T2D patients [22]
		5. Humans (T2D)	5. Plasma	5. LCAC most associated with developing T2D [43]
		6. Humans (gestational and T2D)	6. Serum	6. Strongest association with MCAC [44]
Sepsis/Septic shock	Mitochondrial dysfunction linked to organ failure High energy requirements lead to catabolism Immune response relies on carnitine homeostasis	1. Humans (sepsis)	1. Plasma	1. Increased C2, SCAC, MCAC in non-survivors [45,46]
		2. Humans (septic shock)	2. Serum	2. Increased LC, C2, C3, C8 in non-survivors [47]
		3. Humans (septic shock)	3. Plasma	3. Increased C2, C4 associated with 28-day mortality [48]
		4. Humans (ICU,60/90 with sepsis)	4. Plasma	4. Increased C3, C4, C5, C6 associated with 28-day mortality [49]
Cancer	Reprogrammed metabolism invokes metabolic flexibility Warburg effect leads to upregulated oxidation of FA and BCAA Cell death → release of LC into the blood	1. Humans (HCC)	1. Serum	1. Increased LC, LCAC; decreased SCAC, MCAC [50,51,52,53]
		2. Humans (breast)	2. Plasma	2. Increased C2 associated with disease risk [54]
		3. Humans (colorectal)	3. Serum	3. Predominantly increased SCAC, MCAC, LCAC [55]
		4. Humans (thyroid)	4. Serum	4. Increased MCAC, LCAC [56]
Heart Failure	Cardiac tissue heavily relies on FAO → tissue damage leads to abnormal FAO	1. Humans (HF)	1. Plasma	1. Increased LCAC associated with worse disease severity [57]
		2. Humans (HF)	2. Plasma	2. Increased MCAC, LCAC associated with all-cause mortality and hospitalization [58]
		3. Humans (HFrEF)	3. Plasma	3. Increased C2, SCAC, MCAC, LCAC; C2 and MCAC associated with disease severity [59]

SCAC = short-chain acylcarnitines, MCAC = medium-chain acylcarnitines, LCAC = long-chain acylcarnitines.

**Table 2:** Examples of drugs known to cause alterations in mitochondrial metabolic function.

Drug Name	Valproic Acid (VPA)	Clofazamine (CFZ)	Zidovudine (ZDV)	Cisplatin (CSP)	Propofol	Cyclosporine (CyA)
Chemical Structure	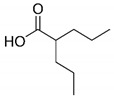	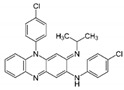	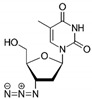	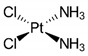	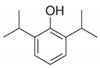	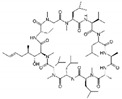
Drug Class	antiepileptic	antibiotic	antiviral	anticancer	anesthetic	immunosuppressant
Mechanism of Pharmacological Action	Increases brain concentrations of gamma-aminobutyric acid (GABA), blocks voltage-gated ion channels, and inhibits histone deacetylase [83]	Inhibits mycobacterial growth and binds preferentially to mycobacterial DNA [84]	Inhibits HIV’s reverse transcriptase (RT) via DNA chain termination [85]	Binds to genomic DNA in the cell nucleus to form cross-links which trigger cytotoxic processes [86]	Increases GABA-mediated inhibitory function in the CNS [87]	Exact mechanism unknown, but thought to inhibit production and release of interleukin-2 [88]

**Table 3:** Examples of drugs that influence carnitine and acylcarnitines levels in the body.

Drug	Proposed Mechanism	Subject	Biospecimen	Drug-Induced Alterations in Carnitine/Acylcarnitine Levels
Valproic Acid	VPA depletes LC by forming valproylcarnitine that is renally eliminated	1. Rats	1. Serum, muscle, and urine	1. Decreased LC (serum, muscle); increased pooled ACs and AC/LC ratio (serum, muscle); increased ACs (urine) [89]
		2. Mice	2. Whole liver	2. Decreased LC; increased AC/LC ratio [90]
		3. Humans (Pediatric)	3. Plasma	3. Decreased LC, increased AC/LC ratio [91]
Clofazimine	CFZ stimulates FAO, decreases urine excretion of LC precursors (methionine, ascorbate)	1. Mice	1. Urine, whole blood	1. Decreased LC (urine); increased LC (whole blood) [92]
Zidovudine	Non-competitively inhibits OCTN2, the primary carnitine transporter Disruptions to mitochondrial respiratory enzymes	1. C2C12 cells 2. Humans	1. Cells 2. Muscle biopsy	1. Decreased LC [93] 2. Decreased LC [94]
Cisplatin	Cytotoxicity → cell death → release of LC into blood Impaired uptake and reabsorption via OCTN2	1. Humans (Various cancers)	1. Plasma, urine	1. Increased LC (plasma); increased renal excretion of LC (urine) [95]
		2. Humans (Various cancers)	2. Serum, urine	2. Increased LC (serum, urine) [96]
Propofol	Disruptions to the mitochondrial ETC Increased permeability of IMM Inhibition of respiratory complexes	1. Humans 2. Humans 3. Humans	1. Serum 2. Plasma 3. Plasma	1. Increased C3-DC, C5 [97] 2. Increased MCAC [98] 3. Increased C4 [99]
Cyclosporine	CyA induced nephrotoxicity impacts carnitine reabsorption ROS generation impairs mitochondrial membrane potential	1. Rats	1. Urine, blood, liver, kidney, pancreas	1. Decreased LC (urine, blood); increased LC (liver); no change (kidney, pancreas) [100]
		2. Humans	2. Serum	2. Increased LC, SCAC [101]

SCAC = short-chain acylcarnitines, MCAC = medium-chain acylcarnitines, LCAC = long-chain acylcarnitines.
